# Exploring the Relationship Between Farmland Management and Manure-Derived Antibiotic Resistance Genes and Their Prevention and Control Strategies

**DOI:** 10.3390/antibiotics14111117

**Published:** 2025-11-05

**Authors:** Chengcheng Huang, Yuanye Zeng, Fengxia Yang, Qixin Wu, Yongzhen Ding

**Affiliations:** 1 Agro-Environmental Protection Institute, Ministry of Agriculture and Rural Affairs, Tianjin 300191, China; huangcheng0206@foxmail.com (C.H.); zengyuanye@foxmail.com (Y.Z.); 2 Dali, Yunnan, Agro-Ecosystem, National Observation and Research Station, Dali 671004, China; 3 College of Resources and Environmental Engineering, Guizhou University, Guiyang 550025, China; qxwu@gzu.edu.cn

**Keywords:** farmland cultivation mode, tillage methods, fertilization management, antibiotic resistance genes, prevention and control strategies

## Abstract

**Background/Objectives:** The application of manure introduces antibiotic resistance genes (ARGs) into farmland, posing a significant public health risk. While tillage and fertilization practices are known to influence soil ecosystems, a systematic synthesis of how tillage patterns specifically regulate the fate of manure-derived ARGs is lacking. **Methods:** This review bridges this critical knowledge gap by systematically analyzing the interactions between conventional/conservation tillage and the distribution, persistence, and transmission of these ARGs. **Results:** It is observed that conservation tillage (e.g., no tillage), while beneficial for soil health, can lead to ARG accumulation at the soil surface, potentially increasing runoff risks, whereas conventional tillage promotes vertical mixing and dilution. A key unique contribution of this review is the systematic comparison of conventional versus conservation tillage, revealing quantitative reductions in ARG abundance. under practices like no till or deep plowing. **Conclusions:** We further con-solidate and propose integrated management strategies, combining precision agriculture, optimized fertilization, and scientific soil management, to mitigate ARG pollution. This work provides a targeted framework for developing more effective intervention measures to ensure agricultural sustainability and safeguard human health.

## 1. Introduction

In modern agriculture, particularly in intensive livestock farming, antibiotics are extensively used for the treatment of animal diseases [1,2]. Nevertheless, the long-term, frequent, and sometime excessive use of antibiotics exerts strong selective pressure on microbial communities, accelerating the evolution and enrichment of antibiotic-resistant bacteria (ARB) and antibiotic resistance genes (ARGs) [3]. These resistant microorganisms and their genetic determinants persist in animal hosts and are subsequently excreted into the environment through animal feces [4,5,6]. Livestock manure, a rich source of organic fertilizers due to its high organic matter and nutrient content [7], also contains residual antibiotics, large numbers of resistant bacteria, and ARGs. When used as fertilizer, it becomes a major route for ARGs to enter and contaminate agricultural soils [8]. These soils then transform into significant reservoirs and hotspots for ARG dissemination [9,10]. The accumulation and spread of ARGs in agricultural ecosystems threaten not only environmental health but also human health via food chain contamination and environmental exposure, posing challenges to public health safety [11]. Rapid, field-deployable biosensors for food-borne pathogens—such as a dual-mode platform enabling ultrasensitive *Salmonella* detection with portable temperature- and pressure-based readouts—offer actionable surveillance options along the food chain [12]. Given their environmental persistence and harmfulness, ARGs are increasingly recognized as emerging environmental contaminants of global concern.

Agricultural tillage practices—ranging from conventional plowing to conservation methods such as reduced tillage and no tillage—significantly alter soil structure, porosity, moisture conditions, organic matter content and distribution, nutrient availability, and microbial community composition and activity [13,14]. Consequently, different tillage practices profoundly influence the spatial distribution, migration, transformation, persistence, and potential horizontal gene transfer of manure-derived ARGs in soils. Research has demonstrated that soil management practices can reshape the resistome structure of soils and potentially promote the horizontal transfer and dissemination of ARGs [15]. Thus, elucidating the collaborative mechanisms by which manure inputs and tillage practices regulate the environmental behavior of ARGs within agricultural ecosystems is essential. Such understanding is critical for assessing associated environmental risks, developing effective strategies for resistance control and mitigation (e.g., optimizing tillage and manure management), and addressing potential public health challenges. However, most studies examine tillage [13,14] or manure-derived ARGs [7] in isolation. A synthesis that integrates how modifiable field practices jointly shape the fate and transport of manure-borne ARGs remains scarce. In particular, the field still lacks a systematic analysis of how tillage, crop rotation, fertilization, and pesticide application restructure soil physicochemical niches and microbial networks to govern colonization, persistence, and dissemination of ARGs.

To address this gap, this review systematically explores the intricate interactions between tillage practices and manure-derived ARGs, shedding light on their distribution, transmission, and persistence in agricultural soils. By synthesizing current research, it elucidates how different tillage methods reshape soil microbial communities and influence ARG dynamics, while also examining the role of manure characteristics, soil properties, and broader agricultural management practices. The review further evaluates mitigation strategies to curb ARG spread and identifies critical gaps in understanding, offering insights into future research directions aimed at promoting sustainable agriculture and safeguarding public health.

## 2. Diversity and Risk of ARGs in Livestock Manure

### 2.1. Diversity of ARGs in Livestock Manure

Animal manure exhibits significant richness and diversity of ARGs [7]. Multiple studies using various methods have consistently revealed this pattern across different animal manure samples. In terms of quantity, a large number of unique ARG types or subtypes have been detected [16]. For instance, one research effort managed to identify as many as 80 different ARGs in dairy cattle manure [17], while another study recognized 48 ARGs belonging to 7 major categories in layer chicken manure [18]. In various animal manure samples (e.g., from pig, sheep, and cow), 16 different ARGs were targeted and detected [19], and yet another study quantified 38 different ARG subtypes in pig slurry and poultry litter manure samples [20]. Studies on soils with long-term manure application indirectly confirmed this finding, with up to 81 unique ARGs [21] or even 234 subtypes [22] detected. Research using high-throughput qPCR techniques further detected 151 [23] to 185 ARGs [24] in manure-related systems.

Secondly, regarding the range of ARGs, those in manure cover almost all clinically important antibiotic classes [7]. Common resistance categories include tetracyclines (such as *tet*-ARGs), sulfonamides (*sul*1, *sul*2), β-lactams (various *bla* genes, including ESBL and carbapenemase genes) [25], aminoglycosides [26], macrolides-lincosamides-streptogramins B (*erm*-ARGs) [18], quinolones [25], and polymyxins [27]. Additionally, genes encoding multidrug resistance (MDR) efflux pumps [28], novel variants of known ARGs [17], and resistance genes for “last-resort” antibiotics critical to human medicine (such as *bla*_NDM_, *van*A, *mec*A) [29] have been discovered. This widespread presence of diverse ARGs in livestock manure poses potential risks to public health and the environment, highlighting the need for further research and management strategies.

This high ARG diversity is not limited to specific animal types but is universally present in manure from major livestock including cattle, swine, and poultry, indicating that this is a common characteristic of animal manure [30]. However, manure from different sources may exhibit variations in specific ARG composition and dominance profiles, potentially reflecting differences in animal species and management practices [31]. For example, studies have found differences in detection frequencies of certain ARGs (such as *tet*C, *tet*M) between poultry manure and swine slurry, and different manure showed different impacts on soil ARG relative abundance [19], although some research indicates that pig manure may lead to higher levels of tetracycline resistance genes [32]. Specific studies of different animal manures (dairy cattle [17], pigs [33]) have demonstrated their unique dominant ARG profiles. Even in bioaerosols generated from manure application, the dominant ARG categories emitted varied by manure type [20]. These differences highlight that while various manures are sources of diverse ARGs, their specific resistance risk characteristics differ. Furthermore, recent meta-analyses synthesizing global data have confirmed the widespread prevalence of ARGs in livestock manure, often identifying core resistomes (e.g., *tet* and *sul* genes) and highlighting geographical hotspots linked to regional antibiotic usage and farming intensities [34]. These large-scale studies reinforce the role of animal manure as a critical node in the global dissemination of antibiotic resistance.

### 2.2. Potential Risks of Manure-Derived ARGs

Antibiotic resistance genes in animal manure present multifaceted potential risks to the environment and public health [35]. When this manure is applied to agricultural fields as fertilizer, it becomes the primary pathway for ARGs, ARBs, antibiotic residues, and potentially co-occurring heavy metals (such as copper and zinc added to feed) to enter soil ecosystems [36]. This not only directly contaminates farmland and alters the original soil “resistome” [37], but pollutants can also enter rivers, lakes, and other water bodies through surface runoff or percolate to groundwater through leaching [36], threatening water resources.

ARGs exhibit significant persistence in the environment, potentially remaining in soils and sediments for months or longer [38]. Even if the original bacteria in manure die, ARGs can continue to exist by transferring to indigenous microorganisms or as extracellular DNA (eARGs) [37]; these eARGs remain biologically active and can be assimilated by environmental bacteria through natural transformation [39]. Additionally, heavy metals commonly found in manure, due to their inherent persistence and frequent co-location with ARGs on the same mobile genetic elements (MGEs) such as plasmids [7], can continue to exert selective pressure on ARGs through co-selection or cross-selection mechanisms even after antibiotic concentrations decrease, extending their survival time in the environment [36].

Manure and its contaminated environments (such as soil) are considered “hotspots” for HGT of ARGs [40]. High-density, diverse bacterial communities, abundant nutrients, and selective pressures from residual antibiotics and heavy metals collectively create ideal conditions for gene exchange [21]. ARGs efficiently spread among different bacteria, including indigenous microorganisms, through MGEs such as plasmids (like IncP-1, IncN, IncW types), integrons (especially class I integrons associated with sulfonamide resistance), and transposons [41]. The main HGT mechanisms include conjugation, transformation, and transduction [7]. Notably, manure not only introduces ARGs into the environment but also introduces numerous MGEs (the “mobilome”), collectively enhancing the propagation potential of ARGs in the environmental microbiome [25]. Bacteriophages as potential ARG vectors add another layer of complexity to the spread of resistance genes [42]. This introduction and spread of ARGs can alter the structure and function of soil and aquatic microbial communities, potentially affecting normal ecosystem service functions such as nutrient cycling [36].

**Figure 1:**
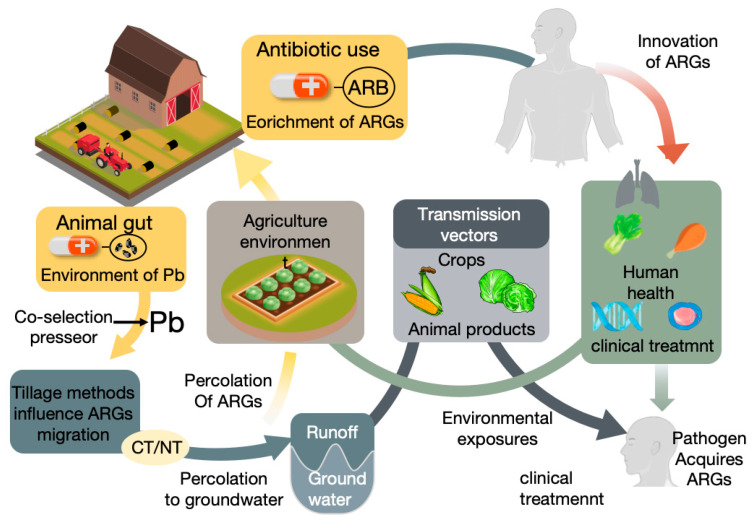
Possible transmission routes of ARGs in the food chain. Antibiotic use in agriculture leads to the enrichment of ARGs and ARB in animal guts. And ARGs can be transmitted to the agricultural environment through manure. Co-selection pressure from pollutants like lead (Pb) further drives ARGs proliferation. However, Tillage methods impact ARGs migration in soil. ARGs can permeate into groundwater and spread via runoff. In the agricultural environment, ARGs are transmitted to humans through crops, animal products, and environmental exposure. They also reach clinical settings, where pathogens acquire ARGs, posing risks to human health. This figure highlights the interconnected pathways of ARGs transmission from farms to humans.

The risks of manure-derived ARGs to public health are primarily manifested in the diversity of human exposure pathways and their contribution to the global antibiotic resistance crisis [43]. Humans can be exposed to ARGs and ARBs from manure through multiple routes. The most direct is through the food chain: consuming animal products (meat, eggs, milk) contaminated during slaughter and processing [44], or consuming fresh agricultural products (vegetables, fruits) grown in manure-amended soil, irrigated with contaminated water sources, or directly exposed to manure [36]. Raw vegetable consumption poses a particular exposure risk [45]. Additionally, environmental exposure is an important pathway, including direct contact with contaminated soil or animal manure (especially important for farm workers and nearby residents) [44], inhalation of farm aerosols or dust containing ARBs/ARGs, and contact with contaminated water bodies through drinking or recreational activities [46]. These exposures may lead to colonization by ARBs or direct infection [44,47]. The interconnections and convergence of these multiple exposure routes toward human hosts are visually synthesized in Figure 1.

A more profound public health concern is that ARGs from the environment (enriched by manure) may transfer to human commensal microbiota or pathogens [48]. Once human pathogens acquire these resistance genes, especially those conferring resistance to clinically important antibiotics (such as penicillins, tetracyclines, sulfonamides, fluoroquinolones, etc.) [25], they will severely undermine the therapeutic efficacy of antibiotics, making infections more difficult to treat, prolonging illness, increasing hospitalization rates and medical costs, and even leading to increased mortality [49]. This not only threatens individual health but also challenges modern medical procedures that rely on effective antibiotics (such as surgery, cancer chemotherapy, organ transplantation, etc.) [50].

Although epidemiologically tracing specific clinical resistant infections directly to a particular manure source is often very difficult, substantial evidence indicates that manure application significantly increases the abundance and diversity of ARGs (including clinically relevant ARGs) in the environment (especially agricultural environments) [51], thereby increasing the probability of these genes eventually entering human pathogens and exacerbating overall public health risks. Therefore, the environmental transmission of ARGs from animal manure is widely recognized as an important factor in exacerbating the global antimicrobial resistance (AMR) crisis [44]. AMR has become a major global public health challenge, projected to cause millions of deaths and enormous economic losses annually if not controlled [52]. This problem clearly demonstrates the close connection between human health, animal health, and environmental health, highlighting the necessity of adopting a “One Health” approach for integrated management and intervention [53]. Manure management is not merely an agricultural waste management issue but a key control point in breaking the AMR transmission chain. The interconnectedness of this chain, as highlighted by studies [7] which show how manure connects antibiotic use and environmental pollution at the animal end to ultimate human health impacts, aligns with the principles of a ‘One Health’ approach.

## 3. Influence of Tillage Mode on the Fate of ARGs in Manure-Amended Soils

### 3.1. Tillage-Induced Soil Structural Changes and Their Impact on ARG Fate

Conventional tillage (CT), characterized by intensive mechanical disturbance and soil inversion such as plowing and rotary tillage, inevitably destroys the soil’s original natural structure, despite aiming to create a loose seedbed for crop growth [54]. CT significantly disrupts soil aggregates, especially the large aggregates crucial for good soil structure, leading to reduced quantity and stability, which consequently exposes soil particles and increases the risk of wind and water erosion [55]. Although CT may temporarily reduce surface soil bulk density and increase porosity [56], long-term or improper operations (e.g., under excessively wet conditions) tend to form a compacted plow pan at the bottom of the tilled layer, restricting vertical water and air movement and hindering deep root penetration. Hydrologically, the degraded soil structure under CT (especially decreased surface aggregate stability) readily leads to surface crusting upon rainfall impact, reducing soil infiltration capacity [57]. Coupled with the obstruction by the plow pan, CT-treated fields often exhibit higher surface runoff and soil loss [57]. Chemically, CT’s plowing action mixes surface crop residues, applied manure, and other organic materials throughout the entire tillage layer [58]. This mixing promotes soil-air contact, accelerating organic matter decomposition and mineralization [55]. While releasing nutrients short-term, this often leads to a gradual decline in surface soil organic carbon (SOC) content over time [55], resulting in the physical incorporation of these surface-accumulated components deeper into the tilled layer, thereby reducing distinct surface enrichment [59]. These tillage-induced alterations to the soil physical and biochemical environment directly influence the fate of ARGs. As evidenced by the data in Table 1, the distinct soil conditions created under different wheat-cultivation modes are associated with significant differences in the abundance and profile of ARGs, highlighting the profound impact of tillage practices on the soil resistome.

In stark contrast, conservation tillage systems (including No tillage, reduced tillage, etc.) aim to minimize soil mechanical disturbance while retaining substantial crop residue on the surface [60]. NT involves minimal disturbance only in narrow planting rows [55], RT employs less frequent or shallower tillage than CT, and Subsoiling Tillage (ST) combines features of both. Conservation tillage, especially long-term NT/RT, significantly improves soil aggregate formation and stability (particularly large surface aggregates) by reducing mechanical disruption [55], which helps maintain pore continuity and preserve large biopores [57]. Soil bulk density under conservation tillage is complex; the surface layer might have higher bulk density than freshly tilled CT soil due to natural settlement and lack of plowing [55,61]. However, the improved soil structure, such as enhanced aggregation and aeration, often observed under long-term NT/RT, contributes to better overall soil physical quality [62], suggesting that bulk density alone is not a comprehensive indicator. Hydrologically, conservation tillage generally performs better. Surface residue cover effectively buffers raindrop impact and protects soil structure [63], while stable structure and biopores enhance infiltration rates and water holding capacity [55]. Consequently, NT/RT significantly reduces surface runoff and soil erosion [63], although runoff might increase under specific conditions [63]. Chemically, the most distinct feature of conservation tillage is the surface enrichment of SOC and nutrients. Due to the lack of mixing, organic materials primarily decompose and accumulate at the surface [55], leading to significantly higher surface SOC content compared to CT [64]. Total nitrogen, available phosphorus, and potassium also show similar vertical differentiation or “stratification” [65], contrasting with CT’s homogenization [59], and providing a unique surface environment for ARGs.

Manure is an important source and transmission medium for ARGs in agricultural ecosystems [66]. Tillage practices, by altering soil physicochemical and hydrological environments, directly influence the occurrence state, persistence, and migration behavior of ARGs introduced with manure [13]. CT’s plowing action, as demonstrated for other soil-applied substances, likely mixes manure-derived ARGs into deeper soil layers (the plow layer) [59], potentially diluting surface concentrations and expanding their distribution volume and contact with the broader soil microbial community. Conventional tillage (CT) significantly alters the soil’s physical and biochemical environment. Such changes could subsequently influence the occurrence state and persistence of manure-derived ARGs and their microbial hosts [13,67]. In contrast, under no-till (NT) systems where manure is surface-applied without incorporation, as was the case in the study by Meyers et al. (2020) [68], the applied manure and associated ARGs are consequently concentrated at or near the soil surface due to the absence of mechanical mixing [13]. This surface enrichment places ARGs in a microenvironment distinctly different from CT. The abundant surface residue and high organic matter content under NT/RT can influence ARG bioavailability and mobility through adsorption. Furthermore, research has suggested that the stable soil aggregate structure formed under no-tillage/reduced tillage (NT/RT) conditions can provide physical protection for soil microorganisms and the biomacromolecules they produce (such as DNA), reducing their exposure to unfavorable environmental factors and thereby potentially prolonging their persistence in the soil [69]. This protective mechanism may also apply to ARGs and their host bacteria. However, the hypothesis that physical protection extends ARG persistence has been challenged by long-term (>11 years) field trial results. Studies found that long-term NT significantly reduced the total relative abundance of various soil ARGs compared to CT [13]. For example, Wang et al. (2023) [13] reported that after 11 years of no tillage, the abundance of 13 different ARG classes in the 0–20 cm soil layer was significantly reduced, including a 44.87% reduction in β-lactam resistance genes and a 34.64% reduction in sulfonamide resistance genes, compared to conventional tillage. This crucial finding indicates that although short-term physicochemical protection might exist in NT environments, the overall soil ecological environment shaped by long-term NT (including its unique microbial community structure, diversity, and activity) exerts a stronger mitigating effect on ARGs [70]. This mitigation likely stems from improved soil health under long-term NT, which enhances the antagonistic capacity and degradation potential of the indigenous microbial community towards ARGs and their hosts [13]. Therefore, assessing the impact of tillage on ARG persistence must consider the decisive role of long-term biological processes over short-term physical factors.

Tillage practices significantly influence ARG migration pathways and risks. Regarding surface runoff, CT degrades soil structure and reduces infiltration [57], leading to significantly increased surface runoff and soil erosion. Consequently, the risk of ARGs (adsorbed on soil particles or within cells) being transported with eroded sediment into surface waters is extremely high [71]. Conversely, the excellent residue cover and more stable soil structure often found under NT/RT systems effectively reduce total runoff and soil erosion, which in turn limits the transport of soil-bound contaminants like pesticides [63]. This means NT/RT significantly lowers the risk of particulate-bound ARG loss via surface runoff, benefiting water quality protection. However, the situation regarding vertical leaching may differ. While NT/RT reduces surface runoff, it increases water infiltration. The stable macropore networks formed under these systems can act as preferential flow paths [57]. Preferential flow pathways (PFPs) act as conduits for the downward migration of microorganisms (e.g., small host bacteria), allowing them to bypass filtration and adsorption by the soil matrix [72]. This mechanism may likewise accelerate the migration of other soluble substances such as soluble extracellular ARGs. Furthermore, potential surface accumulation of ARGs under various tillage systems warrants attention. Organic inputs, such as manure, are known to elevate surface soil ARG concentrations [73]. While no tillage/reduced tillage (NT/RT) generally curtails total runoff and particulate losses, elevated surface ARGs—particularly dissolved forms potentially mobilized from stratified organic matter—could increase their transport risk in initial runoff. This scenario, where surface enrichment of a substance may enhance its dissolved-phase runoff, conceptually parallels reported increases in dissolved phosphorus loss under certain NT conditions, a tillage practice known to modify solute transport dynamics via runoff [63].

### 3.2. Tillage-Driven Microbial Community Restructuring and ARG Persistence Dynamics

Tillage practices [61], beyond their direct effects on pollutant behavior via physicochemical changes [59], exert profound indirect influence by shaping the soil microbiome (community structure, composition, functional capacity), thereby regulating the colonization, persistence, and dissemination potential of manure-derived ARGs [21,74]. Compared to CT’s homogenization effect, conservation tillage (NT/RT) typically leads to SOC accumulation in the surface layer [64]. This enrichment often supports higher microbial biomass (total, bacterial, fungal), particularly in the topsoil [75,76]. Studies report significant biomass increases [77,78], although the effect diminishes with depth [75]. The impact of NT on the fungal-to-bacterial (F:B) ratio is complex. While reduced disturbance under NT has been shown to influence soil microbial communities [13], and theoretically might favor fungal networks, results regarding the F:B ratio are inconsistent [79]. Some studies support a higher F:B ratio under NT [13,72], but a meta-analysis found no significant overall change [79]. This might be related to the typically increased earthworm populations under NT [80]; as ecosystem engineers [81], their interactions with soil microbiota, including fungi [81], can modify soil conditions shaped by tillage.

The impact of tillage on soil microbial alpha diversity (species richness and evenness) appears inconsistent. Some studies report higher diversity under CT, possibly due to new niche creation by plowing [82,83], while others find higher diversity under NT [84], or no significant difference [85], or results varying by year [86]. This suggests the response is strongly influenced by factors like soil type [87], climate [88], tillage duration [83], crop rotation [88], and the long-term, multifaceted impacts of integrated agricultural management practices [82]. However, results for beta diversity (community structure) are more consistent, clearly showing significant differences between CT and NT treatments [82]. Tillage significantly reshapes microbial community composition [89], even if total species number changes are uncertain, because it fundamentally reshapes soil niches [55,62,89,90]. Tillage significantly alters the relative abundance of major microbial groups. Conservation tillage practices, such as no tillage with residue retention, often favor copiotrophic bacteria (e.g., Proteobacteria, Bacteroidetes) adapted to the potentially higher nutrient availability or altered soil environment created by these practices [84]. Conversely, the stable, stratified environment under conservation tillage or no tillage tends to favor certain bacterial groups adapted to specific resource conditions, such as Acidobacteria in some contexts [87], although responses can vary [13]. NT also influences fungal communities, potentially favoring symbiotic fungi like AMF or specific saprophytes [89], and can lead to a greater vertical differentiation of microbial communities compared to conventional tillage (CT), as CT tends to homogenize soil microbial profiles [91]. This shift from copiotrophs to oligotrophs reflects fundamental differences in resource availability and stability imposed by tillage [62,90].

Tillage affects soil enzyme activities involved in element cycling. Due to higher biomass and substrate concentrations, NT surface soils often exhibit higher enzyme activities (e.g., dehydrogenase, phosphatase, some C-cycling enzymes) [60]. However, CT’s aeration and mixing effects can sometimes enhance activity, especially deeper in the profile [92]. Overall microbial metabolic activity also differs; long-term NT is often associated with higher activity [13] (a potential factor in ARG reduction), although CT might stimulate respiration initially [77]. Functional gene profiles reveal shifts: long-term NT shows higher abundance of genes related to C fixation and denitrification (especially at depth) [93], and N fixation (in surface soil) [93], while CT might favor pathways linked to faster nutrient turnover [84,89]. Changes in microbial community structure [79] directly translate into altered functional profiles and process rates [13].

Indigenous soil microbial communities can exert biotic resistance against exogenous microbes, including manure-introduced ARB and their ARGs, inhibiting their colonization [74,94]. Healthier, more diverse, and functionally active communities are generally considered more resistant to invasion [94]. Long-term NT/RT often fosters such communities (higher biomass [79], potentially higher diversity, higher functional activity [13], greater stability [94,95]), thus potentially exhibiting stronger biotic resistance [96]. This aligns with observations of significantly lower ARG abundances under long-term NT [13], suggesting enhanced indigenous community competitiveness limits the establishment of introduced ARB/ARGs. ARG persistence in soil depends on the survival of host bacteria (ARB) and the stability of extracellular ARGs (eARGs) released from dead cells [7], both influenced by microbial activity. Microbial communities mediate eARG degradation via nuclease activity [7]. The potentially higher overall microbial activity in NT surface soils [13] might lead to faster eARG degradation rates; composting studies also link microbial activity to ARG reduction [97]. Competition and predation from indigenous microbes (e.g., fungi [79,98] and earthworms [80,81,99], potentially more abundant under NT) affect ARB survival. The balance between copiotrophic (CT-favored) and oligotrophic (NT-favored) bacteria [90] could also influence persistence. Overall, tillage indirectly affects ARG persistence by altering microbial players and activity levels. The net effect is complex, depending on depth, specific microbial shifts, and soil properties [100,101].

HGT is a primary mechanism for ARG dissemination [102]. Tillage can regulate HGT potential via several microbial mechanisms: (a) Microbial density and spatial structure: NT might increase surface density [79] and stratification [91], potentially boosting local HGT, while its intact fungal networks [13] could facilitate contact. (b) ARG host abundance: CT often enriches Proteobacteria [13,84], known hosts for many ARGs, potentially increasing the soil’s capacity for ARG transfer. (c) MGE abundance and activity: Manure is a major source of MGEs [21]; tillage influences their persistence and transfer efficiency in the soil environment [13]. (d) Microbial network structure: Tillage alters network topology [86]; NT might foster modular networks restricting HGT, while CT’s simpler, denser networks might allow broader transfer [86,103]. Thus, tillage exerts multifaceted control over HGT potential by altering microbial arrangement, composition, and interaction patterns.

### 3.3. Integrated Effects of Tillage–Manure–Soil Interactions on ARG Dynamics

The environmental risk posed by ARGs originating from manure applied to agricultural land is not solely determined by the tillage method employed, but rather stems from the complex interplay between the chosen tillage system, the specific characteristics of the applied manure, the inherent properties of the soil, and the overarching cropping system [66]. The type of manure, defined by its animal source (e.g., chicken manure [73] or cow manure [104]) and treatment (e.g., composting process [104]), significantly influences the initial load of ARGs introduced into the soil, their diversity, and their patterns of accumulation (intracellular vs. extracellular) [73,104]. For example, composting processes can reduce the abundance of some ARGs through microbial activity and high temperature effects, but may also lead to a relative enrichment of extracellular ARGs (eARGs) as a result of thermal or viral lysis of cells [104]. The application of chicken manure, even if composted at high temperatures, resulted in surface soils exhibiting higher diversity and abundance of ARGs compared to soils with only chemical fertilizers [73]. These manure characteristics interact dynamically with tillage practices. Conventional tillage (CT, e.g., plowing), which involves soil turning, tends to mix manure and associated ARGs throughout the tillage layer, although a long-term study suggests that plowing (PT) may result in higher overall abundance of ARGs compared to no tillage (ZT) [13]. No-till (ZT) or reduced-till (RT) systems, which typically involve surface application, may result in enrichment of ARGs in the soil surface [73], although long-term no till has also been shown to be effective in suppressing the accumulation of antibiotic resistance genes in agricultural soils [13]. Although no/less tillage systems generally reduce soil disturbance, the concentration of ARGs (especially eARGs) in the surface layer may increase the risk of their loss through runoff dissolution or leaching, where the mobility of eARGs is influenced by factors such as their size [105]. It is important to distinguish between intracellular ARGs (iARGs), which are mainly transported by bacterial cells, and eARGs, which are capable of adsorbing to soil particles or being transported with water [104,105], as tillage practices may have different impacts on their respective fate by altering the distribution of ARGs and the physicochemical properties of the soil [13].

Soil type, particularly its texture (clay versus sand content), acts as a significant modulator of these interactions [63,106]. Clayey soils, characterized by higher organic matter content (especially where organic matter can accumulate in the surface layer under conservation tillage practices like no till) and greater surface area [107], generally exhibit stronger adsorption of ARGs (and analogous compounds such as pesticides), potentially increasing their persistence in the soil but concurrently reducing their immediate mobility [108,109]. This mechanism aligns with observations where under certain manure amendment conditions, the carriage of ARGs on vegetables was lower, or exhibited distinct distribution patterns, in silty clay loam soils (rich in clay particles) compared to loamy sand soils, suggesting a greater immobilization potential for ARGs in clayey soils [106,110]. However, under no-till (NT) conditions, clay soils can lead to the development of stable soil macropores, creating pathways for preferential flow and thereby potentially increasing the leaching risk for ARGs (and analogous compounds such as pesticides). In contrast, sandy soils, with their lower adsorption capacity and higher permeability, generally permit greater mobility of ARGs, facilitating their leaching into groundwater and thus posing a more significant environmental risk. This risk of leaching in sandy soils can be exacerbated under NT practices due to improved water infiltration [36,63]. The impact of conventional tillage (CT) on soil disturbance, pollutant loss, and erosion can differ significantly depending on soil texture [111]. Therefore, assessing ARG risk requires considering the specific combination of tillage practice and soil type.

**Figure 2:**
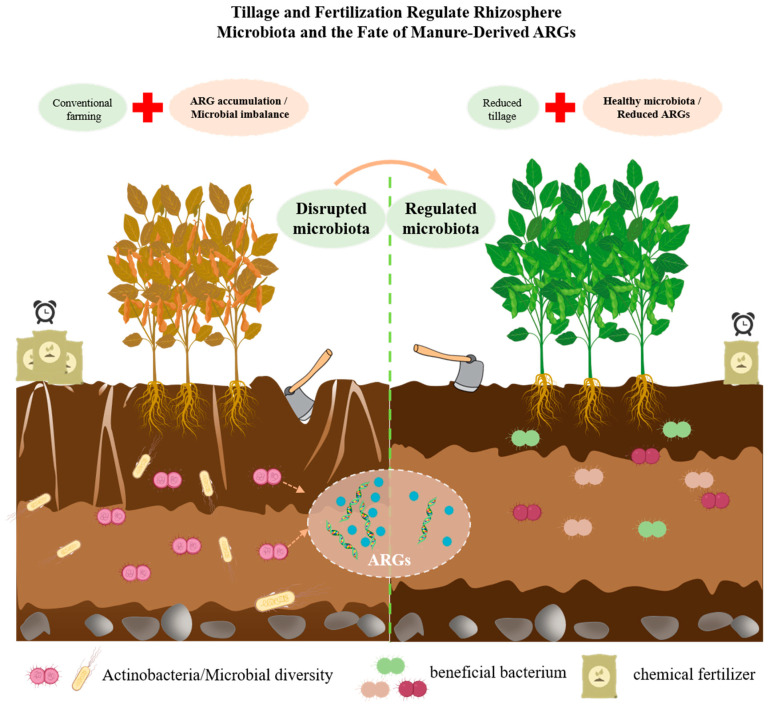
Conceptual illustration of the contrasting impacts of conventional farming versus reduced tillage with precision fertilization on soil microbiota, antibiotic resistance genes (ARGs), and plant health. The left panel depicts conventional farming practices characterized by intensive tillage and excessive fertilizer input, which lead to ARG accumulation, disruption of soil microbial communities, structural degradation of soil, proliferation of harmful bacteria, and impaired plant growth. In contrast, the right panel shows reduced tillage combined with precision fertilization, which fosters a regulated and diverse microbiota, lowers ARG abundance, maintains favorable soil structure, and supports healthier plant development.

Furthermore, the broader cropping system context, including crop rotation and intercropping strategies, interacts with tillage management to influence ARG dynamics [112]. Diversifying cropping systems beyond monocultures, through practices like rotation (growing different crops sequentially) or intercropping (growing multiple crops simultaneously), generally enhances soil health [113]. Crop rotation, particularly involving legumes, can improve soil fertility and structure, and alter soil microbial communities [114]. Intercropping optimizes resource use through niche differentiation and can increase overall biodiversity [115]. Both rotation and intercropping are known to alter soil microbial communities [116]. As detailed in Section 3.2, these shifts in microbial diversity and function are key drivers of ARG persistence. Combining conservation tillage (NT/RT) with such diversified cropping systems may thus foster healthier soil ecosystems, as depicted in the ‘Reduced Tillage with Precision Fertilization’ scenario of Figure 2. Such healthier ecosystems, in turn, could then potentially exhibit greater resilience to ARG accumulation compared to CT monocultures [93].

Comparing different tillage systems reveals distinct ARG risk profiles. The primary risk associated with CT lies in the dissemination of ARGs via soil erosion and subsequent transport of particle-bound ARGs into surface waters. While mixing may initially dilute surface concentrations, the long-term degradation of soil structure poses a significant risk [117]. NT/RT excels at controlling erosion and reducing particle-bound ARG runoff [70]. Long-term NT may eventually suppress ARG accumulation through improved soil health and biological control mechanisms [118]. Strip-tillage (ST) combines in-row tillage with no till and residual mulching between rows, which increases soil temperatures within the strips, while residual stubble between rows helps to retain soil moisture and potentially reduce erosion [119]. Ultimately, the relative risk associated with each tillage system depends heavily on the specific environmental context (soil, climate), manure management practices (type, application method), the chosen cropping system, and the specific ARG risk pathway being prioritized (surface water vs. groundwater).

**Table 1:** The abundance of ARGs in the soil under different wheat-cultivation modes.

Cropping Modes	Provincial Regions	ARG Types	Abundance Levels	Data Source
Wheat–maize rotation	Hebei Province	Multidrug, tetracycline, MLS, and other ARGs	Manure treatment ~2.6 × 10^5^ (RPKM) > Chemical fertilizer treatment ~2.5 × 10^5^ (RPKM) > No fertilizer control ~2.45 × 10^5^ (RPKM)	[120]
Wheat monoculture	Jilin Province	*tetA*, *tetC*, *tetG*, *sul1*, *sul2*, *intI1*	Relative abundance–Total abundance in control soil was ~0.45 (copies/16S rRNA)	[121]
Wheat–soybean rotation	Idaho, USA	*sul1*, *sul2*, *tetW* and other tetracycline, sulfonamide, and macrolide genes	Absolute abundance–Pig manure treatment: sul1 ~10^8^, sul2 ~10^9^, tetW ~10^8^ (copies/g soil)	[66]
Rice–wheat rotation	Jiangsu Province	Total ARGs (especially multidrug resistance genes)	Relative abundance—Fallow treatment: ~1.5 × 10^8^ (copies/g) rotation treatment: ~2.5 × 10^8^ (copies/g)	[122]
Soya, Sunflower, Wheat	Rostov region, RUS	*ermB*, *sul2*, *vanA*	Relative abundance: 10^−7^–10^−2^ (copies/16S rRNA)	[123]
Wheat–Soybean intercropping	Anhui Province	*tetA*	Absolute abundance: Wheat: 5 × 10^4^ (copies/g soil); Soybean: 10^3^ (copies/g soil)	[124]
Eggplant–sweet pepper rotation	Daxing District, Beijing	348 ARGs from 10 major classes	Relative abundance— OF: 0.06203 (copies/16S rRNA); MF: 0.05016 (copies/16S rRNA); IF: 0.04498 (copies/16S rRNA); CK: 0.01978 (copies/16S rRNA)	[9]
Maize–Wheat rotation	Hebei Province	114 ARG subtypes, mainly multidrug, MLSB, aminoglycoside, β-lactam, and tetracycline classes	Relative abundance—M: 0.13; MN: 0.23 (copies/16S rRNA)	[21]
Corn	Lansing, Michigan (MI), USA	89 ARG subtypes, mainly multidrug and tetracycline resistance	Relative abundance: abundance ranged from 0.016 to 0.043 (copies/16S rRNA)	[125]
Rice	Jiangxi Province	144 ARGs subtypes and MGEs	Absolute abundance—no fertilizer: ~3 × 10^6^ (copies/g); swine manure compost: ~6 × 10^6^	[126]
Soybean	Xinjiang Province	289 ARGs subtypes, mainly multidrug and MLS	Relative abundance—plant-derived OMF ~0.046 (copies/cell); chicken manure ~0.052 (copies/cell)	[127]

## 4. Fertilization Management Strategies and Their Impact on Soil ARG Profiles

### 4.1. Differential Effects of Fertilizer Types on ARG Accumulation

Organic fertilizers (livestock manure) and chemical fertilizers have significantly different effects on soil antibiotic resistance groups. Manure application can introduce various ARGs and ARB into soil, resulting from the widespread use of antibiotics in animal husbandry and the excretion of unmetabolized drugs and resistant intestinal microbes [128]. For instance, in China, pig manure has been found to contain dozens of different types of ARGs (conferring resistance to tetracyclines, sulfonamides, and β-lactams) at extremely high abundances [129,130]. In contrast, mineral fertilizers (such as NPK) contain no microorganisms or antibiotics, and therefore do not directly add new ARGs to soil. Field trials indicate that soil treated with raw manure contains significantly higher levels of ARGs than soil treated only with inorganic fertilizers. Reference [66] reported a surge in native β-lactam-resistant bacteria in soil following cow manure application, with no such increase observed with inorganic fertilizers [131]. Notably, this manure-driven enrichment was observed even when using manure from cows that had not received antibiotic treatment, suggesting that factors beyond antibiotic residues (such as nutrient inputs or mobile genetic elements) can stimulate ARG proliferation in soil [21].

Different types of manure (e.g., chicken, pig, and cattle manure) have varying impacts on soil ARG distribution and microbial communities. Generally, poultry and pig manure contribute higher ARG loads than cattle manure, reflecting differences in antibiotic use and feed additives in animal husbandry [132]. In a 130-day microcosm study, Zhang et al. observed that pig and chicken manure caused greater increases in soil ARG diversity and abundance compared to cattle manure, although ARG levels in all manured soils gradually declined from initial peaks over time [133]. Similarly, field analyses found that chicken manure contained approximately 2–4 times more total ARG copies per gram than cattle or sheep manure. These differences are typically associated with more intensive antibiotic use in pig and poultry farming [134]. Additionally, pig and poultry manure often contain high levels of trace metals such as copper and zinc, which are used as feed supplements [129]. These heavy metals persist in manure and act as co-selective agents, enriching metal-tolerant bacteria carrying ARGs. A study by Ji et al. (2012) found that the relative abundance of some specific ARGs was significantly and positively correlated with the concentrations of typical heavy metals in agricultural soils near feedlots in Shanghai, China [135]. For example, the abundance of sulfonamide resistance genes *sul*A and *sul*III showed a strong positive correlation with soil concentrations of copper (Cu) and zinc (Zn) with Pearson correlation coefficients (r) values ranging from 0.78 to 0.87. The results of this study showed that the relative abundance of some specific ARGs was significantly positively correlated with concentrations of heavy metals in agricultural soils near feedlots in Shanghai, China [135]. This co-selective pressure can promote bacteria carrying genetically linked metal and antibiotic resistance determinant clusters, further amplifying genes such as *sul*1 or *tet*C in manure-amended environments.

Pig manure is a known repository of broad-host-range plasmids carrying multiple ARGs. A survey of German pig farms isolated numerous transferable plasmids (IncP-1, IncN, etc.) from manure, with 44 plasmids testing positive for the ampicillin resistance gene *bla*_TEM_ and 68 plasmids testing positive for sulfonamide resistance genes (*sul*1/*sul*2) [41]. These mobile genetic elements facilitate the spread of clinically relevant ARGs from manure to soil bacterial communities. Proper manure treatment before application can mitigate the aforementioned risks. Composted or aged manure significantly reduces the abundance of many ARGs compared to raw manure application [132]. Under suboptimal composting conditions or during cooling phases, surviving resistant bacteria and genetic elements carrying ARGs may proliferate. For instance, another study indicated that composting removed 23–99% of ARGs and integron mobile elements during high-temperature phases, but sulfonamide ARG levels rebounded during the later maturation phase of pig manure composting. Nevertheless, applying well-composted or properly stored manure typically introduces far fewer viable ARB and ARG copies than spreading raw manure, thereby significantly reducing immediate surges in resistance genes in soil environments [132,136].

Long-term fertilization studies indicate that repeated manure applications lead to persistent enrichment of soil ARGs compared to pure chemical fertilizers [7]. In a 15-year field trial, soils receiving annual manure applications accumulated a broader and more abundant resistome, with approximately 81 ARG subtypes and about 0.23 ARG copies per 16S rRNA gene, while soils receiving only chemical fertilizers showed no significant ARG increases [21]. Microbial community structures also changed significantly in manure-amended plots, with variance partitioning analysis showing these community changes as the primary driver shaping soil resistomes. More importantly, long-term manure application significantly increased the proportion of mobile genetic elements involved in horizontal gene transfer [137]. Many ARGs in manured soils were strongly correlated with class 1 integron integrase genes (*intI*1) and transposases, indicating that horizontal transfer facilitated the spread of resistance under chronic manure loads [133,138]. Manure co-factors exacerbated this effect over time: decades of manure application resulted in Cu, Zn, and As accumulation in soils, associated with ARG abundance and maintenance of ARG-metal co-resistance traits [135]. A recent global metagenomic survey further confirmed that organically fertilized soils possess greater diversity of antibiotic and metal resistance genes compared to soils treated solely with inorganic fertilizers [139]. Together, these findings emphasize that long-term use of organic (manure-based) fertilizers can enrich persistent pools of mobile, co-selected ARGs in soil, while strict reliance on chemical fertilizers poses far less risk for environmental antibiotic resistance proliferation.

### 4.2. Effects of Fertilization Timing and Frequency on ARG Dynamics

Frequent and poorly timed manure applications have a pronounced effect on soil microbial communities and ARGs profiles. Manure-fertilized soils tend to harbor more diverse and abundant ARGs than soils receiving only inorganic fertilizer [140]. Long-term field trials demonstrate that repeated annual manure inputs drive cumulative ARG enrichment: a 40-year study reported linear to exponential increases in tetracycline, macrolide, and sulfonamide resistance genes in manured plots [141]. Manure not only introduces resistant bacteria and mobile genetic elements but also selects for indigenous soil bacteria carrying ARGs. Notably, even manure from livestock never given antibiotics can induce a “bloom” of resident antibiotic-resistant bacteria in soil, indicating that nutrient-rich organic amendments alone enrich certain ARG-harboring populations [131]. This shift in the soil resistome underscores that the schedule and type of fertilization critically shape microbial community structure and ARG content over time [21,141].

The timing and frequency of manure applications also influence the persistence of ARGs via residual antibiotics and other selective pressures. Manures often contain drug residues such as tetracyclines and sulfonamides that can persist in soil between applications [132]. For example, pig slurry amendments have been shown to leave measurable concentrations of lincomycin, doxycycline, and tiamulin in soil, which gradually decline over time [142]. Repeated applications at short intervals can lead to the accumulation of such residues, prolonging selection for ARG-maintaining microbes. In a field study, fluoroquinolone and tetracycline residues persisted in sandy soils after successive slurry fertilizations [143]. These lingering antibiotics, along with co-factors like heavy metals from manure, exert continuous selection pressure that favors ARG-bearing organisms [129]. Consequently, genes like sul1 and ermB are often found at elevated levels in soils with frequent manure input [132,142]. Even clinically relevant ARGs such as *bla*NDM-1 and *mcr*-1 have been detected in livestock manures and may persist despite treatment efforts [144].

Excess nutrients and microbial stress from over-fertilization further accelerate HGT. Manure-amended soils show increased levels of class 1 integrons (*intI*1) and plasmid-associated genes, facilitating ARG mobility [41]. Additionally, proliferation of indigenous bacteria carrying β-lactamase genes has been observed following manure application, even without direct antibiotic inputs [131]. Sub-inhibitory antibiotic concentrations, combined with the presence of mobile elements, stimulate genetic exchange and ARG spread. For instance, *mcr*-1 and *bla*NDM-1 increased significantly in soils during composting of contaminated manure, demonstrating HGT potential under manure-induced stress [129,144].

Improper timing of manure application can also increase the risk of ARG transfer to crops. Applying manure shortly before planting or harvest allows ARGs and resistant microbes to persist near root zones and on edible tissues [45]. Similarly, applying raw manure in late fall left elevated ARG levels in soil into the next season compared to spring applications that allowed more time for ARG degradation [140]. These results emphasize the need for sufficient intervals between manure application and crop growth. Current organic farming guidelines recommend a 90–120 day waiting period between raw manure use and harvest, yet recent evidence suggests this may not fully mitigate ARG risk.

Precision fertilization, including strategic fertilizer selection and appropriate manure management, helps control ARG accumulation by reducing nutrient excess and limiting ARG proliferation potential [145]. Field trials show that less frequent manure application, alternating with fallow or chemical fertilizer, allows soil microbial communities time to recover and resistome levels to stabilize. One study showed that after an 11-year cessation of manure input, ARG levels in soil declined but did not fully return to pre-manure baselines [141]. Manure pre-treatment methods—such as aerobic composting, anaerobic digestion, and alkaline stabilization—significantly reduce ARG loads, especially for genes like *erm*B and *tet*W [136,144]. Incorporating treated manure into soil and avoiding surface broadcasting also helps reduce environmental ARG dispersal. Finally, enforcing minimum application-to-harvest intervals and monitoring antibiotic residues in manure provide crucial safeguards to limit ARG transmission through agricultural systems [139].

## 5. Pesticide-Mediated Selection Pressure on Soil Resistome and Agricultural Systems

### 5.1. Pesticide Classes Differentially Modulate Soil ARG Profiles and Microbial Ecology

Different pesticide types exert varied but significant effects on soil microbial communities and the distribution of ARGs [146]. Organophosphorus (OP) insecticides like dimethoate can initially reduce microbial populations [147], while others like methamidophos might stimulate growth [148] or enrich for specific degrading bacteria [149]. OPs are broadly implicated in contributing to antimicrobial resistance (AMR) [150], potentially selecting for multi-resistant bacteria [151] and promoting the transfer of ARG-carrying plasmids [152], plausibly favoring resistance to β-lactams, aminoglycosides, and tetracyclines which are often associated with mobile genetic elements (MGEs) [153]. Broad-spectrum fungicides such as carbendazim and mancozeb can significantly decrease populations of bacteria, fungi, and functionally important groups like nitrogen fixers [152,154], while also acting as co-selectors for ARGs [123]. Studies link these fungicides to increased resistance to tetracyclines [155] and sulfonamides (*sul*1, *sul*2) [123]. Mancozeb and other fungicides can also enhance the horizontal transfer of ARGs [156]. Herbicides like glyphosate show variable impacts on overall microbial community structure [157], but consistently increase the prevalence of ARGs and MGEs [158]. Pesticides exposure can select for bacteria resistant to antibiotics like streptomycin and ampicillin [159] and significantly enhances ARG transfer via conjugation, likely by increasing bacterial cell membrane permeability [158]. Across these classes, concerns exist about multidrug resistance and ARG mobilization via MGEs, alongside complex impacts on crop health balancing pest/disease/weed control benefits [160] against potential phytotoxicity or disruption of beneficial soil functions. These agronomic impacts on the primary crop recipients of pesticide applications are detailed in Table 2.

### 5.2. Dose-Dependent Effects of Pesticides on ARG Dissemination and Crop Performance

Pesticide concentration critically modulates their impact on ARG dynamics and crop health [171,172]. Higher concentrations generally exert stronger selective pressure, potentially increasing the abundance of resistant bacteria and associated ARGs, including sulfonamide and tetracycline resistance genes linked to fungicide residues. Even sub-lethal concentrations can provoke stress responses and select for resistance [123]. Concentration also influences ARG mobility via HGT; specific moderate concentrations of herbicides (e.g., 2–20 mg/L) [158] and organophosphates (e.g., 100 µg/L) have been shown to enhance plasmid conjugation frequency, likely through mechanisms like increased membrane permeability or oxidative stress [152]. However, this effect might be non-linear, as very high toxic concentrations could inhibit the processes required for HGT [173]. Crop health impacts are also dose-dependent, with higher pesticide concentrations increasing the risk of phytotoxicity, growth reduction, and yield loss. This is exemplified by the greater inhibitory effect of higher carbendazim–mancozeb concentrations on soil microbes [154] and dose-dependent growth inhibition in wheat by dimethoate [172]. Balancing the concentration needed for effective pest control [174] against the risks of phytotoxicity and ARG proliferation presents a significant challenge, emphasizing the need for careful dose management and strategies to minimize pesticide reliance.

## 6. Coping Strategies and Future Perspectives

In conclusion, this review systematically synthesized the complex relationship between farmland management and manure-derived ARGs. Our primary finding is that tillage practices act as a critical, yet complex, regulator of ARG fate. While long-term conservation tillage generally fosters a soil microbiome less conducive to ARG persistence compared to conventional tillage, it concurrently creates a risk of ARG enrichment at the soil surface, potentially accelerating their transport via runoff. This highlights that no single tillage method is universally optimal. The significance of this synthesis lies in demonstrating that effective ARG mitigation cannot rely on isolated interventions. Rather, it demands an integrated ‘One Health’ approach, where tillage systems, manure pre-treatment (e.g., composting or anaerobic digestion), and precise fertilization are strategically combined to break the transmission chain from farm to environment. Understanding these intricate interactions is paramount for developing sustainable agricultural policies that ensure both food security and public health.

### 6.1. Coping Strategies: Mitigating ARG Spread in Agricultural Systems

**Figure 3:**
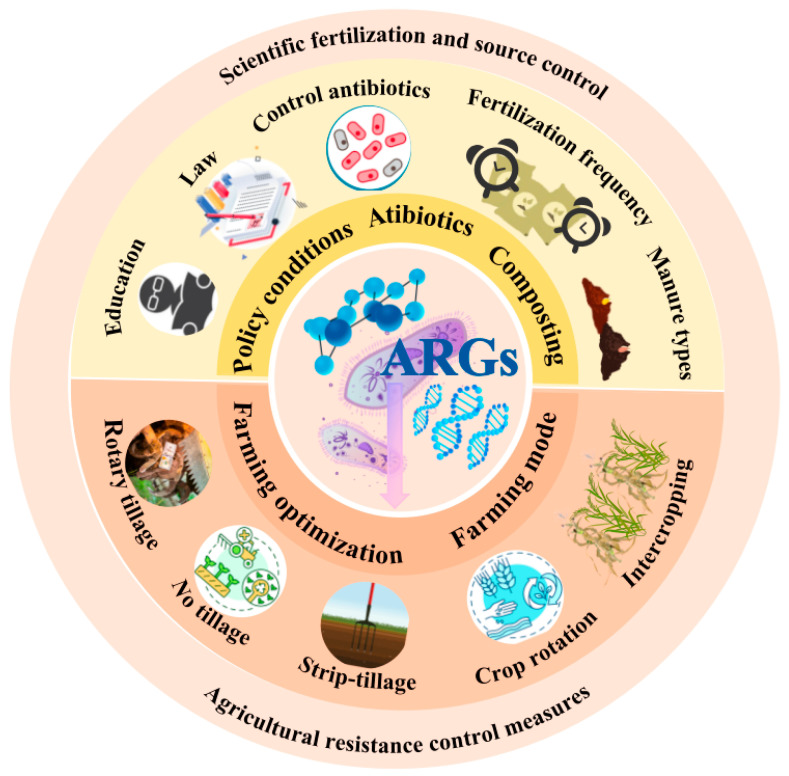
Schematic representation of agricultural strategies for mitigating the spread of antibiotic resistance genes (ARGs). The upper section highlights scientific fertilization and source control approaches, including optimized fertilization frequency, selection of manure types, composting practices, antibiotic regulation, and supportive policy and educational measures. The lower section illustrates agronomic resistance control measures, encompassing tillage practices (rotary tillage, no tillage, strip tillage), crop rotation, and intercropping, which collectively contribute to farming.

A comprehensive suite of strategies to mitigate the dissemination of ARGs within agricultural systems is visually summarized in Figure 3. Effective management of the dissemination of ARGs within agricultural systems necessitates comprehensive strategies. Primarily, it is crucial to recognize that organic fertilizers, particularly animal manure, constitute a principal source of soil ARGs, generally exerting a greater impact than chemical fertilizers. Although the latter have a less direct effect, their excessive application can still indirectly promote resistance by altering soil microbial communities [9]. Consequently, pivotal mitigation measures encompass optimizing fertilization practices and enhancing manure management. Precision fertilization technologies, which apply nutrients on the basis of need, have the potential to reduce total inputs of fertilizers (including organic amendments). However, their widespread adoption is hampered by challenges such as cost, technical complexity and data interpretation [175]. For manure, effective pre-application treatment is crucial. Anaerobic digestion (AD), particularly thermophilic AD or AD combined with pre-treatment methods, generally demonstrates superior efficacy in reducing ARG copy numbers compared to conventional aerobic composting (AC) [176]. Nevertheless, neither AD nor AC can completely eliminate the associated risks; residual ARGs and mobile genetic elements (MGEs) may still enter the soil via treated end-products. Notably, foodborne pathogens harboring heat-resistance determinants—exemplified by the locus of heat resistance (LHR)—can survive otherwise lethal thermal treatments and display cross-protection against oxidative sanitizers (e.g., chlorine), indicating that heat- or disinfectant-based interventions alone may not fully mitigate residual public-health risks [177]. Ensuring the stability of treatment efficacy and controlling MGEs remain persistent challenges [16]. Furthermore, field management practices play a significant role. Crop rotation and intercropping can increase biodiversity, improve soil health and reduce reliance on chemical pesticides and herbicides [178]. While conservation tillage benefits soil and water conservation and soil carbon sequestration [179], the resultant enrichment effect in the surface soil layer may inadvertently create “hotspots” for ARG persistence and transfer, necessitating careful consideration in conjunction with manure management strategies. Management practices during fallow periods and the timing of manure application also influence the residual risk of ARGs [180]. Finally, promoting the widespread adoption of these technologies and management measures critically depends on increasing farmer and public awareness of ARG risks, providing sufficient education, training, and technical support, establishing effective policy incentives, and fostering collaboration among research institutions, industry, and government bodies [181].

### 6.2. Future Perspectives: Research Directions and Challenges

Looking ahead, continuous scientific innovation and international cooperation are indispensable for the effective management of ARGs in agriculture. Research priorities should include the further development and optimization of manure treatment technologies. This involves exploring the synergistic effects of high-temperature composting with additives like biochar to enhance ARG removal efficiency [182], as well as investigating advanced AD pre-treatment (e.g., thermal hydrolysis, microwave) and post-treatment (e.g., digestate composting, advanced oxidation) strategies aimed at achieving more stable and thorough removal of ARGs and MGEs, requiring comprehensive assessment of their environmental and economic viability [183]. To address this global challenge while ensuring food security, it is imperative to vigorously develop and promote alternative strategies that reduce reliance on antibiotics and pesticides [184]. For instance, Li et al. (2023) reported an on-site, CRISPR/dCas9-enabled bimodal biosensor (SCOUT-dCas9) that couples LAMP with dCas9 pull-down to yield fluorescent (SYBR Green I) and colorimetric (ALP/pNPP) readouts, achieving ultrasensitive *Salmonella typhimurium* detection (≈1 CFU mL^−1^) with self-validation across dual channels and successful tests in real food samples [185]. These include bio-ecological pest and weed management, efficient nutrient management technologies, alternatives to veterinary antibiotics, and the genetic improvement of stress-resistant crop varieties [186]. Given the transboundary nature of the ARG issue, strengthening international cooperation in research, policy coordination, knowledge sharing, and global governance is crucial [187]. Additionally, leveraging multidisciplinary knowledge from ecology, genetics, environmental science, and data science to develop precise models capable of predicting ARG dissemination and evolutionary trends will facilitate a shift from reactive responses to proactive prevention and adaptive management, providing robust tools for risk assessment and decision support [188]. Ultimately, controlling ARGs in agriculture demands a systemic approach, integrating source control, process management, ecological regulation, and socio-economic factors within a “One Health” framework to ensure agricultural sustainability and the health of humans and the environment.

## Figures and Tables

**Table 2:** Effects of different pesticides on crops.

Pesticide Type	Representative Compound	Mode of Action	Effect on Crops	Reference
Herbicide	Glyphosate	Inhibits EPSP synthase, blocks aromatic amino acid synthesis	Suppresses plant growth; inhibits root development; affects rhizosphere microbes	[161,162]
Herbicide	Phosphinic acid	Inhibits glutamine synthetase	Disrupts nitrogen metabolism, affects plant growth	[163,164]
Insecticide	Organophosphates	Inhibits acetylcholinesterase, interferes with neural transmission	May affect crop physiological functions	[165,166]
Insecticide	Pyrethroids	Disrupts sodium channels, affects nervous system	May have toxic effects on crops	[167,168]
Fungicide	Benzimidazoles	Inhibits microtubule formation, disrupts cell division	Suppresses fungal growth, protects crops	[163]
Fungicide	Triazoles	Inhibits ergosterol synthesis, disrupts cell membranes	Suppresses fungal growth, protects crops	[169,170]

## Data Availability

No new data were created or analyzed in this study. Data sharing is not applicable to this article.
